# A phylogeny of species near *Agrostis* supporting the recognition of two new genera, *Agrostula* and *Alpagrostis* (Poaceae, Pooideae, Agrostidinae) from Europe

**DOI:** 10.3897/phytokeys.167.55171

**Published:** 2020-11-20

**Authors:** Paul M. Peterson, Steven P. Sylvester, Konstantin Romaschenko, Robert J. Soreng, Patricia Barberá, Alejandro Quintanar, Carlos Aedo

**Affiliations:** 1 Department of Botany MRC-166, National Museum of Natural History, Smithsonian Institution, Washington DC 20013-7012, USA; 2 College of Biology and the Environment, Nanjing Forestry University, Long Pan Road No. 159, Nanjing, 210037, China; 3 Department of Africa and Madagascar, Missouri Botanical Garden, St. Louis, Missouri 63110-2291, USA; 4 Herbarium MA, Unidad de Herbarios, Real Jardín Botánico, Consejo Superior de Investigaciones Científicas, 28014, Madrid, Spain; 5 Department of Biodiversity and Conservation, Real Jardín Botánico, Consejo Superior de Investigaciones Científicas, 28014, Madrid, Spain

**Keywords:** *Agrostis*, *Agrostula*, *Alpagrostis*, classification, ITS, *Neoschischkinia*, plastid DNA sequences, phylogeny, *Podagrostis*, taxonomy

## Abstract

Based on a molecular DNA phylogeny of three plastid (*rpl32-trnK*, *rps16* intron, and *rps16-trnK*) and nuclear ITS regions investigating 32 species of Agrostidinae, we describe two new genera, *Agrostula***gen. nov.** with a single species and *Alpagrostis***gen. nov.** with four species; provide support for five species in a monophyletic *Podagrostis*; and include a small sample of 12 species of a monophyletic *Agrostis* s.s. (including the type and most species of *Neoschischkinia*), that separates into two clades corresponding to A.subg.Agrostis and A.subg.Vilfa. *Agrostula* differs from *Agrostis* in having leaf blades with pillars of sclerenchyma which are continuous between the adaxial and abaxial surface of the blades, dorsally rounded glumes with blunt to truncate and erose to denticulate apices, florets ½ the length of the glumes, lemmas equally wide as long, widest at (or near) apex, apices broadly truncate, irregularly 5 to 7 denticulate to erose, awnless, anthers longer than the lemmas, and rugose-papillose caryopses. *Alpagrostis* differs from *Agrostis* in having geniculate basally inserted awns and truncate lemma apices with lateral veins prolonged from the apex in (2)4 setae. The following eight new combinations are made: *Agrostulatruncatula*, Agrostulatruncatulasubsp.durieui, *Alpagrostisalpina*, Alpagrostisalpinavar.flavescens, *Alpagrostisbarceloi*, *Alpagrostissetacea*, Alpagrostissetaceavar.flava, and *Alpagrostisschleicheri*. In addition, we provide a key separating *Agrostula* and *Alpagrostis* from *Agrostis* s.s. and other genera previously considered as synonyms of *Agrostis*; lectotypify *Agrostisalpina* Scop., *A.schleicheri* Jord. & Verl., *A.truncatula* Parl., and A.truncatulavar.durieui Henriq.; and neotypify *A.setacea* Curtis.

## Abstract (sp)

Sobre la base de una filogenia molecular de ADN de tres regiones plastidiales (*rpl32-trnK*, *rps16* intrón y *rps16-trnK*) e ITS nuclear de 32 especies de Agrostidinae, describimos dos nuevos géneros, *Agrostula***gen. nov.** con una sola especie, y *Alpagrostis***gen. nov.** con cuatro especies; mostramos el apoyo para las cinco especies dentro de Podagrostis monofilético; e incluimos una pequeña muestra de 12 especies de *Agrostis* s.s (que incluye el tipo y la mayoría de las especies de *Neoschischkinia*), este último dividido en dos subclados que corresponden a A.subg.Agrostis y A.subg.Vilfa. *Agrostula* se diferencia de otras especies de *Agrostis* por tener láminas foliares con haces de esclerénquima continuos entre las superficies adaxial y abaxial de los limbos, glumas de dorso redondeado y ápice embotado a truncado y eroso a denticulado, antecios de ½ de la longitud de las glumas, lemas tan anchas como largas, lo más ancho en o cerca del ápice, ápices anchamente truncados, irregularmente 5 a 7 denticulados o erosos, sin arista, anteras más largas que los lemas y cariopsis rugosa-papilosa. *Alpagrostis* se diferencia de otras especies de *Agrostis* por tener aristas geniculadas insertas basalmente y ápices de lema truncados con venas laterales que se prolongan en (2)4 arístulas apicales. Presentamos las siguientes ocho nuevas combinaciones: *Agrostulatruncatula*, Agrostulatruncatulasubsp.durieui, *Alpagrostisalpina*, Alpagrostisalpinavar.flavescens, *Alpagrostisbarceloi*, *Alpagrostissetacea*, Alpagrostissetaceavar.flava y *Alpagrostisschleicheri*. Además, proporcionamos una clave que separa *Agrostula* y *Alpagrostis* de *Agrostis* s.s. y otros géneros previamente considerados como sinónimos de *Agrostis*, lectotipificamos *Agrostisalpina* Scop., *A.schleicheri* Jord. & Verl., *A.truncatula* Parl. y A.truncatulavar.durieui Henriq. y neotipificamos *A.setacea* Curtis.

## Introduction

The genus *Agrostis* L. includes ca. 224 species worldwide and is placed in subtribe Agrostidinae Fr., supersubtribe Agrostidodinae Soreng, tribe Poeae R.Br., and supertribe Poodae L. Liu in subfamily Pooideae Benth. (Soreng et al. 2017). The length of the palea was recognized by Trinius (1820, 1824) as an important character in separating species of *Agrostis* into two groups, those with short paleas in A.sect.Trichodium (Michx.) Trin. and those with longer paleas in A.sect.Vilfa (Adans.) Roem. & Schult. The term “Trichodium net”, based on observations of the Swedish scientist T. Vestergren, to describe the lemma epidermis of *Agrostis* which bear a fine-meshed network when observed under high magnification, is found almost exclusively in those species with short paleas (Björkman 1960).

A detailed review of the infrageneric classification of the *Agrostis* was given by Björkman (1960) and later summarized in Widén (1971), Romero García et al. (1988a), and Saarela et al. (2017). In the former Soviet Union, Tzvelev (1976, 1983) recognized four sections in the genus: Agrostissect.Agrostis (now = A.sect.Vilfa s.s. due to type conservation of *Agrostis*) containing species with long paleas 1/2–2/3 the length of an usually unawned lemma; A.sect.Pentatherum (Nabel.) Tzvelev with long paleas 2/3–1 the length of a dorsally awned lemma; A.sect.Agraulus (P. Beauv.) Tzvelev with short paleas <1/3 the length of a dorsally awned lemma, and; A.sect.Trichodium (Michx.) Dumort. with paleas absent or short <1/6 the length of a usually unawned lemma. Romero García et al. (1988a, b) in the Iberian Peninsula divided *Agrostis* into two subgenera: A.subg.Zingrostis A.T. Romero García, G. Blanca López & C. Morales Torres containing species that have diffuse panicles with widely spreading, capillary and divaricate branches, and paleas 1/2–1 the length of an unawned lemma; and A.subg.Agrostis consisting of three sections, A.sect.Agrostis [= A.sect.Trichodium, A.sect.Agraulus (P. Beauv.) Tzvelev] with paleas <1/3 the length of the lemma; A.sect.Vilfa with paleas 1/2–2/3 the length of a usually unawned lemma; and A.sect.Aperopsis Asch. & Graeb. [= *Neoschischkinia* Tzvelev] with paleas <1/6 the length of the awned or unawned lemma, and an annual lifecycle.

*Podagrostis* (Griseb.) Scribn. & Merr. was initially described as a section of *Agrostis* (Grisebach 1852) and was recently updated and revised by Sylvester et al. (2019a, b, 2020) to include ten species native to the western hemisphere. Five additional species of *Agrostis* were transferred into *Podagrostis* in Sylvester et al. (2020) of which *P.bacillata* (Hack.) Sylvester & Soreng and *P.rosei* (Scribn. & Merr.) Sylvester & Soreng are newly included in our molecular analysis using nuclear internal transcribed spacer (ITS) and three plastid DNA (*rpl32-trnK*, *rps16* intron, and *rps16-trnK*) markers. Previously, *P.thurberiana* (Hitchc.) Hultén was included in a phylogenetic study based on morphology and three plastid regions, and the taxon was weakly supported as the sister group of a strongly supported *Agrostis* + *Polypogon* Desf. clade (Soreng et al. 2007). A limitation of that analysis was that only a single species was included for each of the three genera. No molecular study has included more than two species to test the monophyly of this putative lineage (Saarela et al. 2017). The salient characters separating *Podagrostis* from *Agrostis* are: a) floret usually equaling or subequaling the glumes, sometimes slightly shorter but reaching past ¾ the length of the glumes, b) palea well-developed, usually reaching from (2/3) ¾ to almost the apex of the lemma, c) presence of a glabrous or distally hairy rachilla extension emerging from under the base of the palea as a slender short stub up to 1.4 mm long (rudimentary in most florets of *P.rosei*), and d) lemmas unawned or with a short straight mucro 0.2–0.6 mm long, inserted medially or in the upper half of the lemma, not surpassing the glumes (awn 1.6–2 mm long, inserted in lower 1/3 of lemma, straight or geniculate and usually not surpassing glumes in *P.rosei*) [Sylvester et al. 2020].

Sáez and Rosselló (2000) described *Agrostisbarceloi* L. Sáez & Rosselló from the northern mountains of Mallorca (Balearic Islands) placing it in the *Agrostisalpina* Scop. complex along with *A.curtisii* Kerguélen and *A.schleicheri* Jord. & Verl. These four species share the following synapomorphies: geniculate basally inserted awns and truncate lemma apices that bare lateral setae (extension of the lateral veins) [Romero García et al. 1988a, b; Sáez and Rosselló 2000]. Other species of *Agrostis* with truncate lemma apices include: *A.nebulosa* Boiss. & Reut. [= *Neoschischkinianebulosa* (Boiss. & Reut.) Tzvelev], *A.reuteri* Boiss., *A.truncatula* Parl. (these three placed in A.subg.Zingrostis), *A.pourretii* Willd. (placed in A.sect.Aperopsis), and *A.tenerrima* Trin. (placed in A.sect.Agrostis) [Romero García et al. 1988a, b].

*Neoschischkinia*Tzvelev (1968) initially included two species [*N.elegans* (Thore) Tzvelev = *Agrostistenerrima* Trin., and *N.nebulosa* (Boiss. & Reut.) Tzvelev = *Agrostisnebulosa* Boiss. & Reut.] characterized by having diffuse, open panicles with divaricate and capillary branches, trapezoid lemmas with truncate apices, and caryopses with transverse furrows (Tzvelev 1968). Valdés and Scholz (2006) transferred three more species into *Neoschischkinia* [*N.reuteri* (Boiss.) Valdés & H. Scholz = *A.reuteri*, *N.truncatula* (Parl.) Valdés & H. Scholz = *A.truncatula*, and *N.pourretii* (Willd.) Valdés & H. Scholz = *A.pourretii*].

The main goals of this study were to estimate the phylogenetic relationships of species near or sister to *Agrostis* based on ITS and three plastid DNA regions (*rpl32-trnK*, *rps16* intron, and *rps16-trnK*) for species of Agrostidinae and provide names for two clades that align near but not within *Agrostis* s.s. In addition, we provide a key separating *Agrostula*, gen. nov., and *Alpagrostis*, gen. nov., from *Agrostis* s.s. and other genera considered as synonyms of *Agrostis*. We propose lectotypes for *Agrostisalpina*, *A.schleicheri*, *A.truncatula* and A.truncatulavar.durieui Henriq., and a neotype for *A.setacea*.

## Material and methods

### Phylogenetic analyses

Detailed methods for DNA extraction, amplification, and sequencing are given in Romaschenko et al. (2012) and Peterson et al. (2014, 2015a, b, 2016). We used Geneious Prime 2020 (Kearse et al. 2012) for contig assembly of bidirectional sequences of *rpl32-trnL*, *rps16* intron, *rps16-trnK*, and ITS regions, and Muscle (Edgar 2004) to align consensus sequences and adjust the ﬁnal alignment. We identiﬁed models of molecular evolution for the cpDNA and nrDNA regions using jModeltest (Posada 2008) and applied maximum-likelihood (ML) and Bayesian searches to infer overall phylogeny. The combined data sets were partitioned in accordance with the number of markers used. Nucleotide substitution models selected by Akaike’s Information Criterion, as implemented in jModelTest v.0.1.1, were speciﬁed for each partition (Table 1). The ML analysis was conducted with GARLI 0.951 (Zwickl 2006). The ML bootstrap analysis used 1000 replicates with 10 random addition sequences per replicate. The tree ﬁle from the ML result was read into PAUP where the majority-rule consensus tree was constructed. Bayesian posterior probabilities (PP) were estimated using a parallel version of the MrBayes v3.2.7 (Huelsenbeck and Ronquist 2001; Ronquist and Huelsenbeck 2003) where the run of eight Markov chain Monte Carlo iterations was split between an equal number of processors. Bayesian analysis was initiated with random starting trees and was initially run for four million generations, sampling once per 100 generations. The analysis was run until the value of the standard deviation of split sequences dropped below 0.01 and the potential scale reduction factor was close to or equal to 1.0. The fraction of the sampled values discarded as burn in was set at 0.25.

**Table 1.** Taxon voucher (collector, number, and where the specimen is housed), country of origin, and GenBank accession for DNA sequences of *rps16-trnK*, *rps16 intron*, *rpl32-trnL*, and ITS regions; a dash (–) indicates missing data.

	Taxa	Voucher	Country	rps16-trnK	rps16 intron	rpl32-trnL	ITS
1	*Agrostisalpina* Scop. [= *Alpagrostisalpina* (Scop.) P.M. Peterson, Romasch., Soreng & Sylvester]	Soreng 7484, Gillespie & Peterson (US)	Austria, Niederosterrich	MT410018	–	MT409931	MT396529
2	*Agrostisbacillata* Hack. [= *Podagrostisbacillata* (Hack.) Sylvester & Soreng]	Evans 145, Lellinger & Bowers (US)	Costa Rica, San Jose	MT410019	MT409978	MT409932	MT396530
3	*Agrostisbalansae* (Boiss.) Tzvelev	Soreng 8967b & Cabi (US)	Turkey, Erzurum	MT410020	MT409979	MT409933	MT396531
4	*Agrostiscanina* L.	Herrero 1874, Aedo, Aizpuru, Alarcón, Aldasoro, Castroviejo, Conti, Estébanez, Güemes, Guillén, Navarro, Pedrol, Prunell, Rico, Rodríguez Gracia & Tinti (MA)	Italy, Abruzzo	MT410021	MT409980	MT409934	MT396532
5	*Agrostiscapillaris* L.	Aedo 19209 (MA)	France, Landes	MT410022	MT409981	MT409935	MT396533
6	*Agrostiscurtisii* Kerguélen [= *Alpagrostissetacea* (Poir.) P.M. Peterson, Romasch., Soreng & Sylvester]	Gil s.n. (MA)	Spain, Coruña	MT410023	MT409982	MT409936	MT396534
7	*Agrostiscurtisii* Kerguélen [= *Alpagrostissetacea* (Poir.) P.M. Peterson, Romasch., Soreng & Sylvester]	Louzan s.n. & Rodríguez-Oubiña (MA)	Spain, Coruña	MT410024	MT409983	MT409937	MT396535
8	*Agrostismertensii* Trin.	Smith 1288 (US)	Sweden, Härjedalen	MT410025	MT409984	MT409938	MT396536
9	*Agrostismicrantha* Steud.	Tibet-MacArthur 1516, Wen, Nie, Soreng, Rankin, Yue, Wang & Yue (US)	China, Yunnan	MT410026	MT409985	MT409939	MT396537
10	*Agrostisnebulosa* Boiss. & Reut.	Serra 8114 (US)	Spain	MT410027	MT409986	MT409940	MT396538
11	*Agrostisnervosa* Nees ex Trin.	Soreng 5276, Peterson & Sun Hang (US)	China, Yunnan	MT410028	MT409987	MT409941	MT396539
12	*Agrostispourretii* Willd.	Carrera s.n. (MA)	Spain	MT410029	MT409988	MT409942	MT396540
13	*Agrostisreuteri* Boiss.	Escobar-García s.n. (MA)	Spain	MT410030	MT409989	MT409943	MT396541
14	*Agrostisrosei* Scribn. & Merr. [= *Podagrostisrosei* (Scribn. & Merr.) Sylvester & Soreng]	Peterson 19053 & Sánchez Alvarado (US)	Mexico, Durango	MT410031	MT409990	MT409944	MT396542
15	*Agrostisschleicheri* Jord. & Verl. [= *Alpagrostisschleicheri* (Jord. & Verl.) P.M. Peterson, Romasch., Soreng & Sylvester]	Arán 5627, Patino & Valencia (MA)	Spain, Cantabria	MT410032	MT409991	MT409945	MT396543
16	*Agrostissubpatens* Hitchc.	Lathrop 5571 (US)	Costa Rica	MT410033	MT409992	MT409946	MT396544
17	*Agrostistrachyphylla* Pilg.	Peterson 24374, Soreng & Romaschenko (US)	Tanzania, Kilimanjaro	MT410034	MT409993	MT409947	MT396545
18	*Agrostistruncatula* Parl. [= *Agrostulatruncatula* (Parl.) P.M. Peterson, Romasch., Soreng & Sylvester]	Barberá 916 (MA)	Spain	MT410035	MT409994	MT409948	MT396546
19	*Agrostistruncatula* Parl. [= *Agrostulatruncatula* (Parl.) P.M. Peterson, Romasch., Soreng & Sylvester]	García Río (MA)	Spain, Ciudad Real	MT410036	MT409995	MT409949	MT396547
20	*Agrostistruncatula* Parl. [= *Agrostulatruncatula* (Parl.) P.M. Peterson, Romasch., Soreng & Sylvester]	Morales 2470 (MA)	Spain	MT410037	MT409996	MT409950	MT396548
21	*Calamagrostiscanescens* (Weber ex F.H. Wigg.) Roth	Barta 1999-14 (MA)	Austria, Niederösterreich	MT410038	MT409997	MT409951	MT396549
22	*Calamagrostiscrassiglumis* Thurb.	Howell 23214 (US)	USA	MT410039	MT409998	MT409952	MT396550
23	*Calamagrostisepigeios* (L.) Roth	Calvo 4970 (MA)	Czech Republic, South Bohemian	MT410040	MT409999	MT409953	MT396551
24	*Calamagrostislahulensis* G. Singh	Tibet-MacArthur 1317 (US)	China	MT410041	MT410000	MT409954	MT396552
25	*Calamagrostismacrolepis* Litv.	Soreng 7637, Johnson, Shuvalov, Chapurin, Samsaliev & Samsaliev (US)	Kyrgyzstan, Naryn	MT410042	MT410001	MT409955	MT396553
26	*Calamagrostispseudophragmites* (Haller fil.) Koeler	Cabezas 688, Aedo, Calvo, Castroviejo, Constantidinis, Gonzalo, Güemes, Herrero, Karidas, Medina, Navarro, Pedrol, Prunell, Quintanar, Rico & Rodríguez Gracia (MA)	Greece, Epiro	MT410043	MT410002	MT409956	MT396554
27	*Calamagrostisstricta* (Timm) Koeler	Soreng 7722, Johnson, Shuvalov, Chapurin, Samsaliev & Samsaliev (US)	Kyrgyzstan, Chu	MT410044	MT410003	MT409957	MT396555
28	*Chascolytrumrufum* J. Presl	Butzke 11180 (US)	Brazil	–	–	MT409958	MT396556
29	*Chascolytrumrufum* J. Presl	Wasum 1178 (US)	Brazil, Rio Grande do Sul	–	–	MT409959	MT396557
30	*Chascolytrumsubaristatum* (Lam.) Desv.	Hale 20420 & Soderstrom (US)	Mexico, Chiapas	MT410045	MT410004	MT409960	MT396558
31	*Echinopogoncaespitosus* C.E. Hubb.	Craven 672 (NSW)	Australia, New South Wales	MT410046	MT410005	MT409961	MT396559
32	*Gastridiumphleoides* (Nees & Meyen) C.E. Hubb.	Thomas 9650 (US)	USA	MT410048	MT410007	MT409963	MT396561
33	*Gastridiumphleoides* (Nees & Meyen) C.E. Hubb.	Chase A. 5727 (US)	USA	MT410047	MT410006	MT409962	MT396560
34	*Gastridiumphleoides* (Nees & Meyen) C.E. Hubb.	Gander 8198 (US)	USA	MT410049	MT410008	MT409964	MT396562
35	*Gastridiumventricosum* (Gouan) Schinz & Thell.	Wood 14625 (US)	USA Hawaii, Kaua`i	MT410050	MT410009	MT409965	MT396563
36	*Podagrostisaequivalvis* (Trin.) Scribn. & Merr.	Eastham 10895 (US)	Canada, British Columbia	–	–	MT409966	MT396564
37	*Podagrostisaequivalvis* (Trin.) Scribn. & Merr.	Spellenberg 1574 & Spellenberg (US)	Canada, Alberni-Clayoquot	–	–	MT409967	MT396565
38	*Podagrostishumilis* (Vasey) Björkman	Harrison 10038 (US)	USA, Utah	–	–	MT409968	MT396566
39	*Podagrostishumilis* (Vasey) Björkman	Nielsen 6612 (US)	USA, Utah	–	–	MT409969	MT396567
40	*Podagrostisthurberiana* (Hitchc.) Hultén	Crampton 3860 (US)	USA, California	–	–	–	MT396568
41	*Podagrostisthurberiana* (Hitchc.) Hultén	Davis 2949 (US)	USA, Idaho	MT410051	MT410010	MT409970	MT396569
42	*Podagrostisthurberiana* (Hitchc.) Hultén	Peterson 19755, Saarela & Sears (US)	USA, California	MT410052	MT410011	MT409971	MT396570
43	*Podagrostisthurberiana* (Hitchc.) Hultén	Soreng 7419 & Soreng (US)	USA, California	MT410053	MT410012	MT409972	MT396571
44	*Podagrostisthurberiana* (Hitchc.) Hultén	Terrell 4204 (US)	USA, California	MT410054	MT410013	MT409973	MT396572
45	*Triplachnenitens* (Guss.) Link	Aedo 12786 (MA)	Spain, Murcia	MT410055	MT410014	MT409974	MT396573
46	*Triplachnenitens* (Guss.) Link	López Jiménez 1241 & García Tapia (MA)	Morocco, Nador	MT410056	MT410015	MT409975	MT396574
47	*Triplachnenitens* (Guss.) Link	Rivas Martínez, Costa & Regueiro (MA)	Spain, Islas Baleares	MT410057	MT410016	MT409976	MT396575
48	*Triplachnenitens* (Guss.) Link	Sanz Fábregas s.n. (MA)	Spain, Almeria	MT410058	MT410017	MT409977	MT396576

It is critically important to include the type species of genera and other higher taxa when doing molecular studies to know you are using the name correctly as intended by the original author. The following species are the types of their respective genera and are included in our analyses: *Agrostiscanina* L. (type conserved), *Calamagrostiscanescens* (Weber) Roth, *Chascolytrumsubaristatum* (Lam.) Desv., *Gastridiumventricosum* (Gouan) Schinz & Thell., *Neoschischkiniaelegans* (= *Agrostistenerrima*), *Podagrostisaequivalvis* Trin., and *Triplachnenitens* (Guss.) Link.

Our study was designed to test relationships of three of the four species (*A.alpina*, *A.curtisii*, and *A.schleicheri*) of the *Agrostisalpina* group, all five species that have been attributed to *Neoschischkinia* (*N.elegans*, *N.nebulosa*, *N.pourretii*, *N.reuteri*, and *N.truncatula*), *Podagrostis*, *Gastridium* P. Beauv., *Triplachne* Link, and representative samples of *Agrostis*, *Calamagrostis* Adans., and *Chascolytrum* Desv. All of these genera have been found in a clade in previous molecular analyses and in our unpublished trees investigating a large number of species in *Agrostis*, *Calamagrostis*, *Cinnagrostis* Griseb., and *Koeleria* Pers. (Saarela et al. 2017; Barberá et al. 2019a, b; Peterson et al. 2019). Previous analyses of *Polypogon* found members of the genus nested in a grade within *Agrostis* and there was incongruence between the plastid and nuclear signals (Saarela et al. 2017; Romaschenko et al. unpubl.). We do not address this question here (i.e., *Polypogon* is not included in our sampling) since we lack a large sample of species within *Agrostis* and it is beyond the scope of our study. *Echinopogoncaespitosus* C.E. Hubb. in subtribe Echinopogoninae Soreng was chosen as the outgroup since it lies outside of the Agrostidinae, but inside supersubtribe Agrostidodinae (Soreng et al. 2017; Tkach et al. 2020).

### Taxonomy

Herbarium acronyms follow Index Herbariorum (Thiers, continuously updated). In this treatment glabrous means without pubescence (in the sense of slender, relatively soft hairs). Smooth indicates no prickle-hairs with broad bases and/or hooked or pointed apices (i.e., pubescence can occur on a smooth surface, and a rough or scabrous surface can be glabrous). Specimens in the United States National Herbarium (US) and the Real Jardín Botánico Herbarium (MA) were reviewed for this study, in addition to Romero Zarco (1987), Romero García et al. (1988a, b), Sáez and Rosselló (2000), Clayton et al. (2006), Cope and Gray (2009), and Portal (2009) were consulted during preparation of the descriptions. Beyond types (some only seen in images), only material from herbaria where specimens have been checked and verified by the authors are cited. Parts of the generic key were adapted from Sylvester et al. (2020).

## Results

### Phylogeny

**Figure 1.**
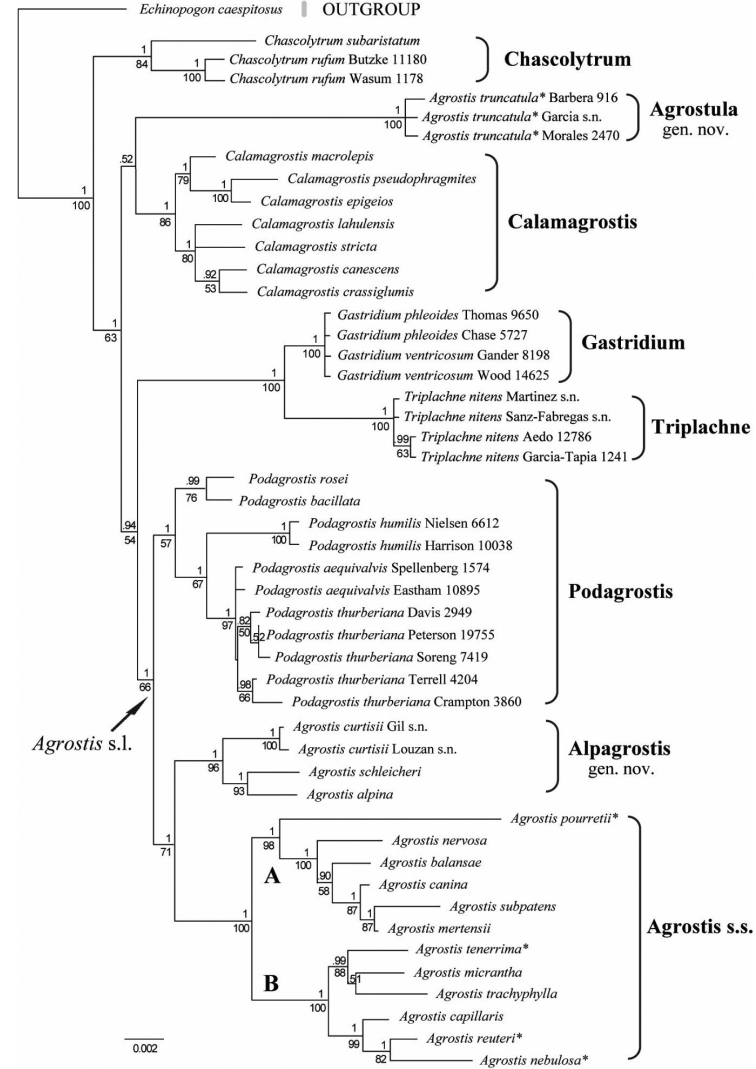
Maximum-likelihood tree inferred from combined plastid (*rpl32-trnL*, *rps16* intron, *rps16-trnK*) and ITS sequences. Numbers above the branches are posterior probabilities; numbers below the branches are bootstrap values; accessions marked with an asterisk* were formerly included in *Neoschischkinia*; and letters refer to clade A = Agrostissubg.Agrostis and clade B = A.subg.Vilfa. Scale bar: 0.002 substitutions per site.

A total of 176 new sequences from 33 species (48 individuals) are reported in GenBank (Table 1). Total aligned characters for individual regions and other parameters are noted in Table 2. The resulting plastid and ITS topologies were inspected for conflicting nodes (see Fig. 1) with ≥ 80% bootstrap support (BS) and/or posterior probabilities (PP) ≥ 0.95. No supported conflict was found so plastid and ITS sequences were combined.

**Table 2.** Characteristics of *rps16-trnK*, *rps16* intron, *rpl32-trnL*, and ITS, and parameters used in Bayesian analyses indicated by Akaike Information Criterion (AIC).

	***rps16-trnK***	***rps16 intron***	***rpl32-trnL***	**Combined plastid data**	**ITS**	**Overall**
Total aligned characters	738	845	904	2487	712	3199
Number of sequences	41	40	47	128	48	176
Likelihood score (-ln*L*)	1259.16	1449.21	1888.70		1989.55	
Number of substitution types	6	6	6	–	6	–
Model for among-sites rate variation	gamma	gamma	gamma	–	gamma	–
Substitution rates						
rAC	2.44683	2.33760	1.03926	–	0.78611	–
rAG	2.12801	1.84060	0.64852		2.03233	
rAT	0.11415	0.31850	0.20833		1.27811	
rCG	1.41016	0.78529	0.73967		0.31482	
rCT	2.47892	2.54521	0.97480		5.07499	
rGT	1.00000	1.00000	1.00000		1.00000	
Character state frequencies						
fA	0.28602	0.35597	0.36767	–	0.22141	–
fC	0.16385	0.15120	0.14893		0.29792	
fG	0.16537	0.18750	0.13618		0.29123	
fT	0.38477	0.30534	0.34722		0.18944	
Proportion of invariable sites	0.37013	0.1041	0.36504	–	0.30563	–
Substitution model	TVM+G	GTR+I+G	GTR+G	–	GTR+I+G	–
Gamma shape parameter (α)	0.90138	0.45913	0.83500	–	0.38018	–

The ML tree from the combined plastid and ITS regions (Fig. 1) is well resolved (posterior probabilities identified in the Bayesian analysis are included on the ML tree, and most clades include a PP = 1), with strong support (BS ≥ 96–100) for the following clades: two species of *Gastridium*, four accessions of *Triplachnenitens*, an *Agrostis* s.s. clade that includes two subclades A and B, three accessions of *Agrostistruncatula*, and the *Agrostisalpina–A.curtisii–A.schleicheri* clade; moderate support (BS = 84–86%) for seven species of *Calamagrostis* and two species of *Chascolytrum*; and weak support (BS = 57%) for five species of *Podagrostis*. *Chascolytrum* is basal followed by, in order of divergence, a clade with *Agrostistruncatula* sister to *Calamagrostis*, a clade with *Gastridium* sister to *Triplachne* which is sister to the remaining species in the *Agrostis* s.l. clade (PP = 1, BS = 66). In *Agrostis* s.l., *Podagrostis* is sister to the *Agrostisalpina–A.curtisii–A.schleicheri* clade and the *Agrostis* s.s. clade.

## Discussion

Our molecular sampling of five species of *Podagrostis* is the largest to date. In an earlier Romaschenko et al. (unpubl.) study of the three species then in the genus, *P.humilis* (Vasey) Björkman exhibited incongruence with the nuclear ITS signal aligning within the *Podagrostis* clade and the plastid signal aligning as sister to *Agrostis* s.s. in a grade with the *Agrostisalpina–A.curtisii–A.schleicheri* clade at the base. The addition of *P.bacillata* and *P.rosei* in our analysis eliminated this anomaly. In an earlier study primarily using different DNA markers with only *P.aequivalvis* and *P.rosei* (as *Agrostisrosei* Scribn. & Merr.), Saarela et al. (2017) found *P.rosei* to be part of a well-supported clade with four Chinese species of *Deyeuxia* Clarion ex P. Beauv. and *Calamagrostisbolanderi* Thurb. + *P.aequivalvis*. Although *C.bolanderi*’s placement in a strongly supported lineage with *P.aequivalvis* provides support for its transfer to *Podagrostis*, we hesitate to include it here because it may represent a separate hybrid between *Podagrostis* and *Calamagrostis* (Sylvester et al. 2020). A robust phylogeny with the inclusion of *P.colombiana* Sylvester & Soreng, *P.exserta* (Swallen) Sylvester & Soreng, *P.liebmannii* (E. Fourn.) Sylvester & Soreng, and *P.trichodes* (Kunth) Sylvester & Soreng is needed, as well as the Asian species of *Deyeuxia* that are allied with the group and are in need of generic realignment.

Affinities of *Agrostistruncatula* are unclear, given the lack of support for its position (PP = 0.52) in the phylogeny sharing a common ancestor with *Calamagrostis* rather than aligning within *Agrostis* s.l. *Agrostistruncatula* has many unique morphological characteristics and differs from other species of *Agrostis* in having the combination of perennial habit, leaf blades with pillars of sclerenchyma that are continuous between the adaxial and abaxial surface of the blades, dorsally rounded glumes with blunt to truncate and erose to denticulate apices, open and diffuse panicles, florets ½ the length of the glumes, lemmas equally wide as long, widest at (or near) apex, apices broadly truncate, irregularly 5 to 7 denticulate to erose, awnless, anthers longer than the lemmas, and rugose-papillose caryopses. We, thus, describe *Agrostula* gen. nov. below based on the single species, *A.truncatula*, with two subspecies. We find no support for recognizing *Neoschischkinia* (Tzvelev 1968; Valdés and Scholz 2006), since four of the five species attributed to the genus align in the *Agrostis* s.s. clade: *A.nebulosa*, *A.reuteri*, and *A.tenerrima* (type of *Neoschischkinia*) in Agrostissubg.Vilfa (clade B), and *A.pourretii* in A.subg.Agrostis (clade A); whereas *A.truncatula* is phylogenetically isolated from Agrostis (Agrostula). All these species exhibit unusual characteristics not commonly found within *Agrostis*, i.e., diffuse, open panicles with divaricate and capillary branches, trapezoid lemmas with truncate apices, and caryopses with transverse furrows. However, without molecular DNA evidence, earlier systematists could not predict the complicated phylogenetic history of *Agrostulatruncatula*.

Our rationale for recognizing the *Agrostisalpina* complex in a new genus, *Alpagrostis* gen. nov., is straightforward. Much like *Podagrostis*, there are salient morphological features, i.e., geniculate basally inserted awns and truncate lemma apices with setaceous lateral veins, and there is strong clade support as sister to *Agrostis* s.s. The branch length of the *Alpagrostis* clade is moderately long indicating genetic differentiation shared among its members separating it from other clades. Sáez and Rosselló (2000) suggested that *Agrostisbarceloi*, a tetraploid (2*n* = 28), is closely related to *A.schleicheri*, a hexaploid (2*n* = 42), and might have originated by the splitting of the shared ancestral lineage. The morphological features shared by *A.barceloi* and *A.schleicheri* suggest they may be derived from the diploids, *A.alpina* (2*n* = 14) or *A.curtisii* (2*n* = 14) since the former two species are geographically and genetically isolated (Sáez and Rosselló 2000). Massó et al. (2016) surveyed 40 of the 100 known individuals of the extremely narrow endemic, *A.barceloi*, for allozyme diversity, showing all loci to be monomorphic or with fixed heterozygosity consistent with allopolyploid origin (interspecific hybridization process and subsequent chromosome duplication) [Stebbins 1947; Crawford 1989; Soltis and Soltis 2000].

The *Agrostis* s.s. clade is divided into two strongly supported A and B clades that correspond to species that align in the Agrostissubg.Agrostis (clade A) or Agrostissubg.Vilfa (Adans.) Rouy (clade B) [≡ A.sect.Vilfa (Adans.) Roem. & Schult.]. As mentioned in the introduction, palea length is an important character used to separate these two subgenera and all species in clade A have paleas ≤1/3 the length of the lemma as expected, sometimes rudimentary or absent as in e.g. *A.mertensii* Trin., *A.subpatens* Hitchc. However, not all species in clade B have paleas ½–2/3 the length of the lemma since *A.tenerrima* has paleas 1/6 the length of the lemma and only about 0.1 mm long. This is not terribly surprising since hybrids among species of *Agrostis* are often fertile, and inter-subgeneric hybrids include *A.canina* × *A.stolonifera* L., a cross between the type of each subgenus of *Agrostis* (Widén 1971; Belanger et al. 2003; Watrud et al. 2004). In the future we intend to publish a large phylogeny of *Agrostis* with a comprehensive species sampling. In this larger paper we will also address the hybrid origins that complicate species relationships in *Agrostis* s.s. with members of *Polypogon*, *Lachnagrostis* Trin., and *Chaetotropis* Kunth, genera that form a clade sister to or are reticulately intermeshed within *Agrostis* s.s. (Saarela et al. 2017; Soreng et al. 2017: 268).

### Taxonomic treatment

#### Agrostula

Taxon classificationPlantaePoalesPoaceae

P.M. Peterson, Romasch., Soreng & Sylvester gen. nov.

4C2FE805-65B8-59BC-BF12-5B3287C017EA

urn:lsid:ipni.org:names:77212587-1

##### Type.

*Agrostistruncatula* Parl.

##### Diagnosis.

The one species of *Agrostula* differs from all other species of *Agrostis* by its glumes being dorsally rounded, not keeled, smooth throughout, and with apices blunt to truncate and erose to denticulate. Further differentiation can be made by the combination of perennial habit, leaf blades with pillars of sclerenchyma that are continuous between the adaxial and abaxial surface of the blades, panicles open and diffuse, florets ½ the length of the glumes, lemmas equally wide as long, widest at (or near) apex, apices broadly truncate, irregularly 5 to 7 denticulate to erose, awnless, paleas c. ½ the length of the lemma, anthers longer than the lemma, caryopsis surface rugose-papillose, and its ecology, being found growing in very shallow soils.

##### Description.

***Perennials*** moderately to densely tufted. ***Culms*** 10–40 cm tall, erect, arching, or geniculate-ascendant, slender, smooth, usually with 3 or 4 nodes extended above the basal foliage. ***Tillers*** intravaginal, extravaginal innovations absent. ***Leaves*** mostly basal, in fascicles of few to many leaves; ***sheaths*** often as long as or sometimes longer than the internodes, glabrous, smooth; ***ligules*** 0.5–4 mm long, longer than they are wide in subsp. truncatula and shorter than they are wide in subsp. durieui, oblong, hyaline, glabrous, smooth, apices truncate to acute, dentate; basal and tiller ligules 0.5–2.5 × 1–2.5 mm; upper culm ligules 3–4 × 1–2.5 mm in subsp. truncatula; ***blades*** flat, conduplicate, or convolute, straight to sometimes recurved after flowering, acute, firm to rigid, glabrous, abaxially scabrous, adaxially scabrous; blades of lower culm and tillers 3–7 cm long, 0.7–2 mm in diameter as flat, folded or rolled; blades of upper culm 1–4 cm long, 0.5–1.2 mm in diameter as flat, folded or rolled. ***Inflorescence*** c. 2–20 × 2–12 cm, a panicle, diffuse and open, broadly ovoid; ***panicle branches*** divaricate, capillaceous, with spikelets present only in the distal 1/3–½, glabrous, smooth; ***pedicels*** generally twice as long as the spikelets or longer, thickened, apices clavate, glabrous, smooth. ***Spikelets*** 1–1.7 mm long, 1-flowered, disarticulating above the glumes, dorsally compressed or very weakly laterally compressed; ***glumes*** equal or subequal, ovoid-lanceolate, membranous, 1-veined, the vein inconspicuous, dorsally rounded, smooth throughout, apices truncate to blunt and minutely notched, erose to denticulate; ***floret*** c. ½ the length of the glumes, sessile; ***lemmas*** 0.5–0.8 mm long in subsp. truncatula and (0.7–)0.9–1(–1.2) mm long in subsp. durieui, broadly ovoid, equally wide as long, widest at (or near) apex, membranous, dorsally rounded, 5-veined, veins usually evident to distinct, with at least the outer veins excurrent, usually glabrous or sometimes pubescent, smooth throughout, apex broadly truncate and denticulate, with the veins terminating in 5 to 7 teeth 1/8–1/5 the length of the lemma, awnless; ***paleas*** 0.3–0.5 mm long, c. ½ the length of the lemma, glabrous, smooth, apices bifid, denticulate; ***calluses*** rounded, blunt, glabrous or almost so, abaxially smooth; ***rachilla*** prolongation absent. ***Flowers*** perfect; ***lodicules*** 0.1–0.3 mm long, c. ½ as long as the palea, 2 in number, acute; ***anthers*** 0.7–1 mm long, 3 in number; ***ovaries*** glabrous. ***Caryopses*** 0.8–1.1 mm long, generally longer than the lemmas, only partially concealed at maturity, ellipsoid, surface rugose-papillose, ventrally sulcate, sulcus distinct, almost without rostellum; hilum narrowly elliptic c. 1/6–1/3 the length of the caryopsis; endosperm liquid. 2*n* = 14 + 0–4B (Garde 1951; Björkman 1960; Fernandes and Queiros 1969; Queiros 1974, 1979; Romero García and Blanca López 1988).

##### Distribution and ecology.

Iberian Peninsula and northern Africa, distributed in France, Spain, Portugal, and Morocco. Found in Mediterranean, Iberian-Atlantic and cold temperate, often high-elevation, environments of the Pyrenees. Forms part of pioneer grassland species assemblages which grow on very shallow and sandy ‘skeleton’ soils, apparently reliant on climatic humidity in addition to precipitation for its water supply. Usually flowering from June to July.

##### Notes.

*Agrostulatruncatula* also differs in its leaf blade anatomy from most other species of *Agrostis* in having pillars of sclerenchyma which are continuous between the adaxial and abaxial surface of the blades. These continuous pillars of sclerenchyma are exceptionally thick and found only on the margins and central vein in subsp. truncatula, while subsp. durieui has thinner continuous sclerenchyma packets in the margins, central and primary veins (Romero García and Blanca López 1988: fig. 4C–F). Costal cells and intercostal long cells of the abaxial blade surface are also distinct, with *A.truncatula* differing from species of *Agrostis* in the Iberian Peninsula in having paired s_0_-z cells in the costal zone, and l_3_ type long cells in the intercostal zone (Romero García and Blanca López 1988). Stomata are also apparently absent on the abaxial blade surface, a character found in only a few other species in the Iberian Peninsula, i.e., *Agrostisreuteri* and *Alpagrostissetacea* (Romero García & Blanca López, 1988).

#### Agrostula truncatula

Taxon classificationPlantaePoalesPoaceae

(Parl.) P.M. Peterson, Romasch., Soreng & Sylvester comb. nov.

AC493EEE-B06E-57B7-8FB2-E4D1903760AB

urn:lsid:ipni.org:names:77212590-1

≡ Agrostistruncatula Parl., Fl. Ital. 1: 185. 1848 ≡ Neoschischkiniatruncatula (Parl.) Valdés & H. Scholz, Willdenowia 36(2): 663. 2006. Type: Spain, Sierra de Guadarrama, Aug 1841, *G. Reuter s.n.* (lectotype, **designated here**: FI-016207 [image!]; isolectotypes, FI-016206 [image!], FI-012389 (Webb herbarium, left hand plant) [image!]).

#### Agrostula truncatula subsp. durieui

Taxon classificationPlantaePoalesPoaceae

(Boiss. & Reut. ex Willk.) P.M. Peterson, Romasch., Quintanar, Soreng & Sylvester comb. nov.

E8B9CD9F-09F4-51E6-896A-FDC015DB6278

urn:lsid:ipni.org:names:77212591-1

≡ Agrostisdurieui Boiss. & Reut. ex Willk., Suppl. Prodr. Fl. Hisp. 15. 1893 ≡ Agrostistruncatulasubsp.durieui (Boiss. & Reut. ex Willk.) Asch. & Graebn., Syn. Mitteleur. Fl. 2(1): 193. 1899 ≡ Agrostisdelicatulasubsp.durieui (Boiss. & Reut. ex Willk.) Rivas Mart., Lazaroa 2: 328. 1980 ≡ Neoschischkiniatruncatulasubsp.durieui (Boiss. & Reut. ex Willk.) Valdés and H. Scholz, Willdenowia 36(2): 663. 2006. Type: Spain. Asturias: Peñaflor [“Hab. in Asturiis freq., usque ad summa juga occident., Peñaflor”], 16 Jun 1835, *M.C. Durieu de Maisonneuve s.n.* [Durieu Plant. Select. Hispano-Lusit sect. 1 Asturicae. Collectae, no. 173] (lectotype, designated by A.T. Romero García and G. Blanca, Taxon 35(4): 695. 1986: P-02219803 [image!]; isolectotypes: P-03487772 [image!], W-18890096450 [image!].
= Agrostisdurieui Boiss. & Reut. ex Gand., Bull. Soc. Bot. France 43: 210. 1896, nom. illeg. hom., non Boiss. and Reut. ex Willk. 1893. Type: Spain. Palencia: m. “Peña Labra, in fissuris, rupium cacuminis, 5700 ft, 26 Jul 1894, *M. Gandoger* s.n.” (lectotype, designated by S. Castroviejo and A. Charpin, Candollea 54(2): 475. 1999: LY [lower specimen]).
= Agrostistruncatulavar.durieuiHenriq., Bol. Soc. Brot. 20: 49. 1903, nom. Illeg. hom., nonsubsp.durieui Asch. & Graebn. Type: Spain. Asturias, 27 May 1864, *Borgeau 2716*. (lectotype, **designated here**: P-03330466 [image!]; isolectotypes: P-02220227 [image!], P-03330465 [image!], P-03487775 [image!].
= Agrostistruncatulasubsp.commista Castrov. & Charpin, Candollea 38(2): 676. 1983, nom. illeg. superfl. Type: Spain. Zamora: Lubián, Chanos, proximidades del puerto de Padornelo, 29T PG 7356, 1200 m, 30 Dec [Jul] 1977, *S. Castroviejo 790* (holotype: MA-242072 [image!]; isotype: G-00191448 [image!]).

##### Notes.

Romero García et al. (1988a) provide a key to differentiate the two subspecies. The typical subspecies has ligules as long or longer than wide with acute apices, conduplicate leaf blades that recurve at anthesis, and shorter lemmas 0.5–0.8 mm long whereas Agrostulatruncatulasubsp.durieui has ligules wider than long with truncate apices, flat, rarely conduplicate leaf blades that do not recurve at anthesis, and longer lemmas (0.7–)0.9–1(–1.2) mm long. Portal (2009) treated subsp. durieui as *Agrostisdurieui* for France, and did not recognize *A.truncatula* as being in France.

#### Alpagrostis

Taxon classificationPlantaePoalesPoaceae

P.M. Peterson, Romasch., Soreng & Sylvester gen. nov.

E50DC2B8-15A1-5239-891A-E7D400634E3E

urn:lsid:ipni.org:names:77212592-1

##### Type.

*Agrostisalpina* Scop.

##### Diagnosis.

The species of *Alpagrostis* differ from *Agrostis* by a combination of characters in having plants densely tufted with only intravaginal innovations, leaves mainly basal, basal leaf blades involute and setaceous or filiform, conduplicate and acute, 0.1–1.2 mm in diameter as folded or rolled, ligules longer than they are wide, spikelets generally > 3 mm long, lemma apices truncate with lateral veins prolonged from the apex in 2 (*A.setacea*) or 4 setae 0.1–0.5 mm long, and, crucially, and lemmas with a well-developed awn, 3–7.4 mm long, inserted basally c. 0.1–0.4 mm from the base of the lemma, conspicuously twisted and geniculate.

##### Description.

***Perennials***, densely tufted. ***Culms*** 4–75 cm tall, erect or slightly geniculate at the base, slender, smooth or scabrous in the upper part, usually with 2–3 nodes extended above the basal foliage. ***Tillers*** intravaginal, extravaginal innovations absent. ***Leaves*** mostly basal, in fascicles of few to many leaves; ***sheaths*** shorter than the internodes, glabrous, smooth or scabrous; ***ligules*** 0.4–5 mm long, longer than they are wide, oblong, hyaline, glabrous, smooth, apices truncate, subacute, acute, entire to dentate; basal and tiller ligules 0.4–3 × 0.15–1.3 mm; upper culm ligules 1.7–5 × 0.7–1.5 mm; ***blades*** involute and setaceous or filiform and acute, tender to firm, straight to recurved, glabrous, abaxially smooth to scabrous, adaxially scabrous; blades of the lower culms and tillers 2–25 cm long, 0.1–1.2 mm in diameter as folded or rolled; blades of upper culm 1.5–10 cm long, 0.2–1.5 mm in diameter as folded or rolled, generally wider and shorter than tillers. ***Inflorescence*** (1.5–)2–15 × 0.5–3.5 cm, a panicle, lax and open to loosely to densely contracted and spikelike; ***panicle branches*** erect, ascendant or patent, with spikelets present from the base to only in the distal ½, glabrous, densely scabrous (or smooth in *A.barceloi*); ***pedicels*** as long as the spikelets, cylindrical, apices clavate, glabrous, densely scabrous (or smooth in *A.barceloi*). ***Spikelets*** (2.7 in *A.barceloi*–)3–5.2(–5.5) mm long, 1-flowered, disarticulating above the glumes, weakly laterally compressed; ***glumes*** unequal, the lower shorter and thinner than the upper, upper glume longer than the length of the floret by c. 0.8–1.9 mm, lanceolate, membranous, glabrous, keel scabrous throughout or in the distal ½, lateral veins smooth or scabrous distally, surfaces smooth or scabrous distally, apices acute or mucronate; lower glume 1-veined; upper glume (1-veined in *A.barceloi*) 3-veined; ***floret*** sessile, much shorter than the glumes; ***lemmas*** (1.8 in *A.barceloi*–)2–3.7, lanceolate, membranous, dorsally rounded, 5-veined, veins usually evident to distinct, with at least the outer veins excurrent, glabrous or thinly pubescent at the base with hairs up to 0.4 mm long, surface smooth to densely scabrous with aculeate (thin short stiff) prickles throughout, apex truncate with lateral veins prolonged from the apex in 2 (*A.setacea*) or 4 setae 0.1–0.5 mm long, awned with awn inserted basally c. 0.1–0.4 mm from the base of the lemma (or sometimes in the lower 1/5–1/4 in *A.barceloi*), awn well-developed, 3–7.4 mm long, surpassing the glumes, geniculate in roughly the middle, distinctly twisted proximally with usually at least 2 full twists below the bend, smooth proximally, scabrous distally or for most of the length; ***paleas*** 0.4–1 mm long, 1/5–1/3 the length of the lemma, glabrous, smooth, apices bifid, dentate, irregularly dentate or emarginate; ***calluses*** rounded, blunt, pilose, with hairs 0.3–0.7 mm long inserted all around or in 2 lateral tufts, abaxially smooth; ***rachilla*** prolongation absent. ***Flowers*** perfect; ***lodicules*** 0.4–0.6 mm long, ½–2/3 as long as the palea, 2 in number, acute to lanceolate; ***anthers*** 0.7–2.3 mm long, 3 in number; ***ovaries*** glabrous. ***Caryopses*** 1.7–2 mm long, shorter than the lemmas, concealed at maturity, ellipsoid or fusiform, surface smooth (becoming narrow and shriveled with age), ventrally sulcate, sulcus distinct, almost without rostellum; ***hilum*** 1/6–1/3 length of the caryopsis, narrowly elliptic; ***endosperm*** liquid. 2*n* = 14 (In *A.setacea*, *A.alpina*), 28 (*A.barceloi*), or 42 (*A.schleicheri*) [Frey 1997; Sáez and Rosselló 2000].

##### Distribution and ecology.

Europe and Mediterranean. Found in cold temperate, often high-elevation environments, often found growing on nutrient poor soils. Usually flowering from June to August.

##### Notes.

All caryopses examined from herbarium specimens had a liquid lipid endosperm or were shriveled with a deep sulcus, implying that fresher specimens likely had a liquid endosperm. Agrostissect.Bromidium (Nees & Meyen) E. Desv. shares many characteristics with *Alpagrostis*, such as lemma apices terminating in scabrous setae, well-developed, thickened, twisted and geniculate awns inserted in the lower 1/3 of the lemma, palea < 1/3 the length of the lemma, caryopses with liquid to semi-liquid endosperm. Based on molecular DNA studies, Romaschenko et al. (unpubl.) and Tkach et al. (2020) found *Bromidium* to align within *Agrostis* s.s.

*Alpagrostisbarceloi* differs somewhat from the other species in the genus, in terms of the panicle branches and pedicels being smooth, spikelets sometimes being shorter, 1-veined upper glumes, and awn sometimes inserted slightly higher up the lemma.

#### Alpagrostis alpina

Taxon classificationPlantaePoalesPoaceae

(Scop.) P.M. Peterson, Romasch., Soreng & Sylvester comb. nov.

B2D35C64-94C1-5B59-85F3-124F9529A24F

urn:lsid:ipni.org:names:77212593-1

**Figure 2.**
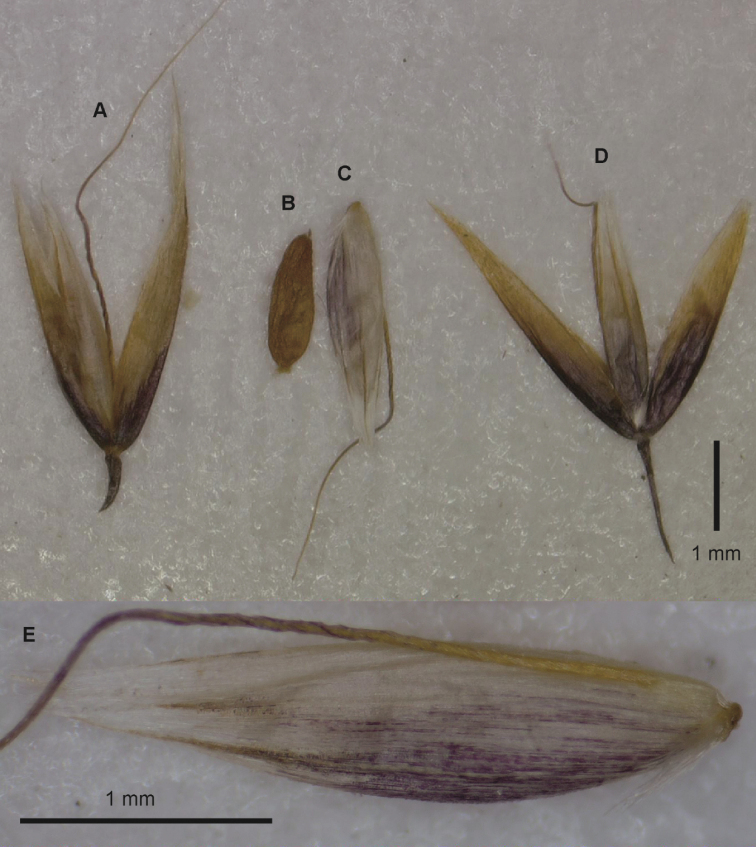
*Alpagrostisalpina***A**, **D** spikelets **B** caryopsis **C** floret **E** floret, showing dorsal surface. Plant fragments taken from *Sain-Lager 3* (US-1628154).

≡ Agrostisalpina Scop., Fl. Carniol. ed. 2, 1: 60. 1772 ≡ Agraulusalpinus (Scop.) P. Beauv., Ess. Agrostogr.: 5. 1812 ≡ Agrestisalpina (Scop.) Bubani, Fl. Pyren. 4: 287. 1901. Type: “Habitat in Alpibus Vochinensibus” and “HALL Hist. n. 1477”, SCHEUCHZ. Gram pag. 140, Prodr. P. 22, tab. 4, fig. 1.”, original material: In siccioribus Alpium Helveticarum & Rhaeticarum pratis, *J. Scheuchzer s.n.* (lectotype, **designated here**: W-18890240472 [image!]. fig. 2

#### Alpagrostis alpina var. flavescens

Taxon classificationPlantaePoalesPoaceae

(Honck.) P.M. Peterson, Romasch., Soreng & Sylvester comb. nov.

7ADD123C-B2F5-50D3-B15F-7B8AA0592C96

urn:lsid:ipni.org:names:77212596-1

≡ Airaflavescens Honck., Gew.: 212. 1782 ≡ Avenaaurata All., Fl. Pedem. 2: 255. 1785, nom. nov. (non Avenaflavescens L.) ≡ Agrostisaurata (All.) Suter, Fl. Helv. 1: 61. 1802, nom. superfl. ≡ Agrostisflavescens (Honck.) Host, Icon. Descr. Gram. Austriac. 4: 52. 1809 ≡ Agrostisrupestrisvar.aurata (All.) Clairv., Man. Herbor. Suisse: 16. 1811 ≡ Avenarupestrisvar.aurata (All.) Clairv., Man. Herbor. Suisse: 16. 1811 ≡ Trichodiumflavescens (Host) Schult., Oestr. Fl., ed. 2, 1: 165. 1814 ≡ Agraulusflavescens (Host) Sweet, Hort. Brit., ed. 2: 556. 1830 ≡ Agrostisalpinavar.flavescens (Honck.) Schrad., in Schlechtendal, Linnaea 12: 435. 1838 ≡ Agrostisalpinavar.aurata (All.) Ducommun, Taschenb. Schweiz. Bot.: 852. 1869 ≡ Agrostisalpinaf.aurata (All.) Beldie, Fl. Reipubl. Popularis Sin. 12: 163. 1972. Type: Switzerland. Bagnes A. Haller hist. 1488 [a description] (lectotype needed).

#### Alpagrostis barceloi

Taxon classificationPlantaePoalesPoaceae

(L. Sáez & Rosselló) P.M. Peterson, Romasch., Soreng & Sylvester comb. nov.

DE6B2A5E-AE95-5579-A796-FE4FF3A1E2D7

urn:lsid:ipni.org:names:77212597-1

≡ Agrostisbarceloi L. Saéz & Rosselló, Bot. J. Linn. Soc. 133: 361–365, f. 1. 2000. Type: Spain. Insulae Balearicae [Balearic Islands], Majorca, in praeruptis rupium umbrosis calcareis septentrionalibus loco dicto Puig Major de Son Torrella, 1400 m, 31SDE8206, 14 Aug 1998, *L. Sáez 5132* (holotype: BC-852322; isotypes: BCC, M, W-20040000640 [image!], herb. L. Sáez).

##### Notes.

This species is included in *Alpagrostis* based on its similar morphology, although this needs to be confirmed in molecular analyses. Certain characteristics sometimes differ from the other species in the genus, i.e., spikelets and lemmas sometimes shorter, insertion of the awn sometimes higher on the lemma, panicle branches and pedicels smooth or scaberulous. *Alpagrostisbarceloi* shares with other member of the genus, conduplicate leaf blades, truncate lemma apices with setaceous extensions of the lateral veins, and ecologically is a strict orophyte, much like *A.alpina* and *A.schleicheri* (Sáez and Rosselló 2000).

#### Alpagrostis setacea

Taxon classificationPlantaePoalesPoaceae

(Poir.) P.M. Peterson, Romasch., Soreng & Sylvester comb. nov.

02AC0B55-4E10-534E-A56E-36EC5812B5A4

urn:lsid:ipni.org:names:77212601-1

**Figure 3.**
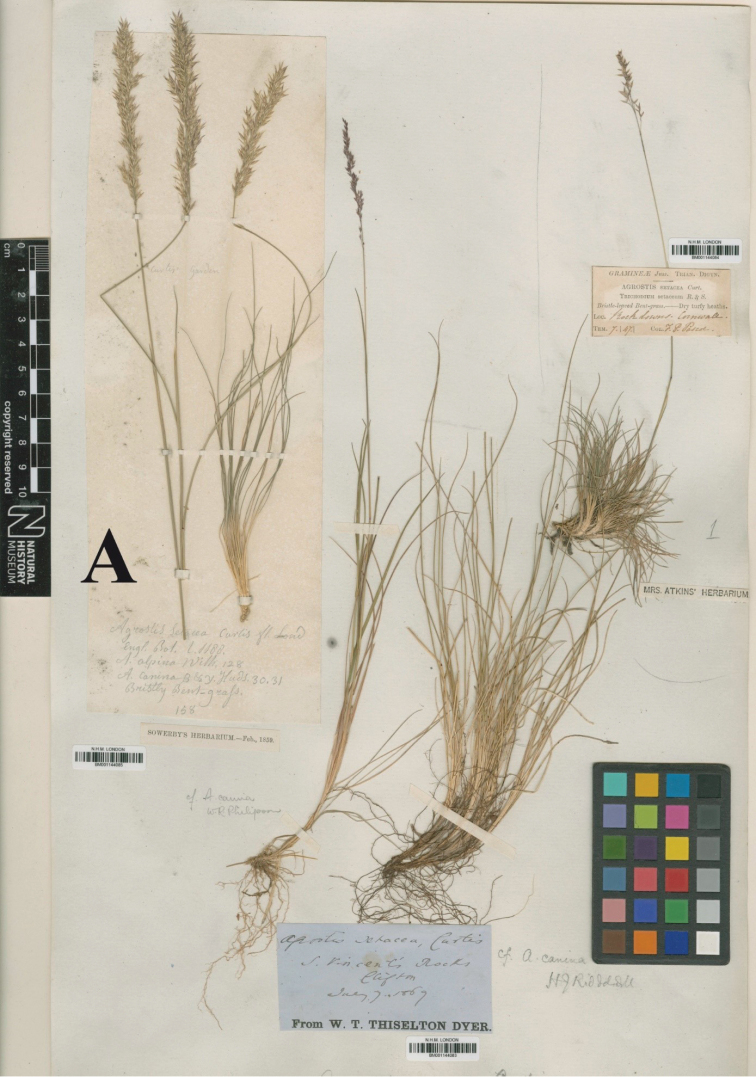
Neotype of *Agrostissetacea* Curtis [= *Alpagrostissetacea* (Poir.) P.M. Peterson, Romasch., Soreng & Sylvester] from Curtis’s garden (BM-001144085), upper left hand specimen indicated by **A**.

≡ Agrostissetacea Curtis, Pract. Obs. Brit. Grasses ed. 1: 35, no. 4. post (Aug) 1787, nom. illeg. hom. (non Villars (Feb) 1787) ≡ Agrostissetacea Curtis, Fl. Londin. 6, t. 12. 1798 ≡ Agrostisrupestrisvar.setacea Poir. in Lam., Encycl., Suppl. 1: 247. 1810 ≡ Vilfasetacea (Poir.) P. Beauv., Ess. Agrostogr.: 16. 148. 1812 ≡ Trichodiumsetaceum (Poir.) Roem. & Schult., Syst. Veg. ed. 15 bis, 2: 280. 1817 ≡ Agraulussetaceus (Poir.) Gray, Nat. Arr. Brit. Pl. 2: 149. 1821 [1822] ≡ Agrestissetacea (Poir.) Bubani, Fl. Pyren. 4: 286. 1901 ≡ Agrostiscurtisii Kerguélen, Lejeunia, n.s., 75 (Err. & Corr.): 1. 1975 ≡ Agrostiscurtisii Kerguélen, Lejeunia, n.s., 75 (Err. & Corr.): 1. 1975. Type: England. Curtis’s garden [Sowerby’s Herbarium], (neotype, **designated here**: BM-001144085 [image!]). fig. 3A.

##### Notes.

Philipson (1937) mentions “No authentic specimens of Curtis have been preserved. There is one specimen in the British Museum Herbarium, originally from “Curtis’s garden” (BM-001144085), which may be taken as representative of the species.” Philipson was possibly referring to this specimen. On the neotype there are three different collections on the same sheet. The specimen on the upper left of the sheet is BM-001144085 (Fig. 2A).

#### Alpagrostis setacea var. flava

Taxon classificationPlantaePoalesPoaceae

(Des Moul.) P.M. Peterson, Romasch., Soreng & Sylvester comb. nov.

FDD74356-6AEE-55D9-B74A-A23D6B90FBC5

urn:lsid:ipni.org:names:77212602-1

≡ Agrostissetaceavar.flava Des Moul., Actes Soc. Linn. Bordeaux 11: 320. 1840 ≡ Agrostiscurtisiivar.flava (Des Moul.) Portal, Agrostis de France: 193. 2009. Type: France. Dans les bois découverts, les bruyères et les landes rases, aux environs de Sagonzac (Périgord), 26 May 1838, *M.C. Durieu de Maisonneuve #90bis* (holotype: not found; isotypes: MPU-027078 [image!], MP-027079 [image!], W-18890240353 [image!], W-18890240354 [image!]).

#### Alpagrostis schleicheri

Taxon classificationPlantaePoalesPoaceae

(Jord. & Verl.) P.M. Peterson, Romasch., Soreng & Sylvester comb. nov.

8D0F2308-6732-5A32-B442-E80D6E49BFE7

urn:lsid:ipni.org:names:77212600-1

≡ Agrostisschleicheri Jord. & Verl., Arch. Fl. France Allemagne 1: **347**, 346–348. 1855 ≡ Trichodiumschleicheri (Jord. & Verl.) Fourr., Ann. Soc. Linn. Lyon, n.s., 17: 181. 1869 ≡ Agrostissubspicata Arv.-Touv., Essai Pl. Dauphiné: 67. 1871, nom. illeg. superfl. ≡ Agrostisalpina proles schleicheri (Jord. & Verl.) Asch. & Graebn., Syn. Mitteleur. Fl. 2(1): 187. 1899 ≡ Agrestisschleicheri (Jord. & Verl.) Bubani, Fl. Pyren. 4: 288. 1901 ≡ Agrostisalpinasubsp.schleicheri (Jord. & Verl.) Rouy, in G. Rouy & J. Foucaud, Fl. France 14: 69. 1913. Type: France. Débris mouvants des rochers calcaries de Mt. St-Nizier près de Grenoble (Isère), 15 Jul 1854, Jean-Baptiste Verlot 1584 (lectotype, **designated here**: P-03161255 [image!], isolectotypes: BM-001134099 [image!], BM-001134098 [image!], MPU-027081 p.p. Verlot 1584 [image!], MPU-027082 [no image], P-03656627 [image!]).

##### Notes.

Jordon (1855) cited the following five collections: the Jura sur le Reculet (Ain), and Mont Ventoux, in August 1841, *A. Jordan*; Mt. St-Nizier near Grenoble, *Verlot*; Bex (canton of Vaud), *E. Thomas*; Mt. St-Nizier near Grenoble, *Clement*. Also cited is a report of Reuter of his collection from Jura sur le Reculet [P-03161256, image!], and *Agrostisfiliformis* sensu *Vill*. We select The *Verlot 1584* specimen as there are several duplicates, and P-03161255 as the lectotype because that sheet is not mounted with any other collection as the MPU and BM sheets seem to be.

### Key to differentiate taxa of *Agrostula* and *Alpagrostis* from *Agrostis* and other genera previously considered as synonyms of *Agrostis* by Watson and Dallwitz (1992) and Clayton and Renvoize (1986)

**Table:** 

1	Spikelets disarticulating below the glumes, the glumes, floret, and part of the pedicel falling together as a unit; glume apices lanceolate or lanceolate-subulate, muticous, mucronate or awned; palea < ½ the length of the lemma	***Polypogon* Desf.**
–	Spikelets disarticulating above the glumes, the glumes remaining on the inflorescence after the florets have fallen; glumes acute to acuminate, not awned; palea of varying length, absent or rudimentary to equaling the length of the lemma	**2**
2	Rachilla extension present (cases where it is sometimes rudimentary key both ways), of varying lengths (sometimes very short, and requiring the base of the palea checked closely to distinguish the structure from hairs), glabrous or pilulose to densely pilose; palea well-developed, generally > 2/3 the length of the lemma	**3**
–	Rachilla extension absent; palea of varying length	**4**
3	Lemmas densely pubescent, with rigid and abundant hairs; callus and rachilla notably hairy; lemmas with a well-developed usually geniculate and twisted awn, > 1 mm long, inserted in the lower or upper half of the lemma, clearly exceeding the glumes; taxa from southern Hemisphere (Australia, Malaysia, New Zealand, South Africa and South America)	***Lachnagrostis* Trin.**
–	Lemmas glabrous; callus and rachilla glabrous or with short hairs emerging from only the rachilla apex and the basal side-ridges of the callus; lemmas unawned or with a short straight awn, usually < 0.5 mm long, inserted in the upper half of the lemma, not or barely exceeding the glumes (awn well-developed, 1.6–2 mm long, inserted in lower 1/3 of lemma, straight or geniculate and usually not surpassing glumes in *Podagrostisrosei* (Scribn. & Merr.) Sylvester & Soreng, but then callus and rachilla glabrous, rachilla very short, < 0.3 mm long, glabrous, plants from Mexico); taxa from North, Central and South America	***Podagrostis* (Griseb.) Scribn. & Merr. (in part)**
4	Lemma apex terminating in 2 or 4 scabrous setae 0.1–2 mm long; lemma with a well-developed geniculate and twisted awn inserted basally or in the lower 1/3 and surpassing the glumes; paleas < 1/3 the length of the lemma; calluses pilulose or densely tufted; leaf blades often filiform or involute; lemma surfaces pilose (*Bromidium*) or usually glabrous (*Alpagrostis*); caryopses with liquid endosperm becoming narrow and shriveled with age	**5**
–	Lemma apex entire or finely dentate with short teeth at the end of each lateral vein; lemmas muticous, with a straight mucron 0.2–1 mm long, or with a long geniculate and twisted awn to 6+ mm long, inserted in the lower, middle or upper 1/3 of the lemma but usually not basally, not surpassing to greatly surpassing the glumes; lemma surface usually glabrous (sometimes pilose e.g. *Agrostiscastellana* L.); calluses usually glabrous or with hairs restricted to lateral lines continuous with the basal lemma margins; leaf blades of various forms but less often filiform or involute; caryopsis usually rounded, with hardened endosperm, less often with liquid endosperm	**6**
5	Anthers 0.2–0.7 mm long; lemma surface often pilose; awn inserted in the lower 1/3 but usually not basally; longest setae of lemma apex 0.4–2 mm long; caryopsis thin or with liquid endosperm; leaf blades filiform or flat, generally 1–4 mm diam.; annuals from southern South America	**Agrostissect.Bromidium (Nees & Meyen) E. Desv.**
–	Anthers 0.7–2.3 mm long; lemma surface usually glabrous or pilulose basally; longest setae of lemma apex 0.1–0.5 mm long; awn inserted basally; leaf blades filiform or involute, 0.1–1.5 mm diam. as folded or rolled; perennials of Europe and NW Africa	***Alpagrostis* P.M. Peterson, Romasch., Soreng & Sylvester**
6	Floret equaling or subequaling the glumes, sometimes slightly shorter but reaching past ¾ the length of the glumes, usually with a short rachilla prolongation emerging behind the palea (sometimes absent in many florets of *P.rosei* and *P.humilis* so check many spikelets); paleas well-developed, usually reaching from (2/3) ¾ to almost the apex of the lemma; lemmas muticous or with a short straight awn 0.2–0.6 mm long, inserted medially or in the upper half of the lemma, not surpassing the glumes (awn well-developed, 1.6–2 mm long, inserted in lower 1/3 of lemma, straight or geniculate and usually not surpassing glumes in *P.rosei*)	***Podagrostis* (Griseb.) Scribn. & Merr. (in part)**
–	Floret notably shorter than the glumes, usually 1/3–3/4 the length of the glumes, rarely longer, without a trace of a rachilla prolongation; paleas well-developed, poorly-developed, or absent, when well-developed reaching from ½–¾ the length of the lemma; lemmas muticous, with a short straight awn 0.2–1 mm long, or with a long geniculate and twisted awn to 6+ mm long, inserted basally, medially or in the upper half of the lemma, not surpassing to greatly surpassing the glumes	**7**
7	Glumes dorsally rounded, not keeled, smooth throughout, apices blunt to truncate and erose to denticulate; palea c. ½ the length of the lemma; panicles open and diffuse; lemmas equally wide as long, widest at (or near) apex, apices broadly truncate, irregularly 5 to 7 denticulate to erose, awnless; anthers longer than the lemma, caryopsis surface rugose-papillose; perennials; growing from very shallow soils; from the Iberian Peninsula and Northern Africa	***Agrostula* P.M. Peterson, Romasch., Soreng & Sylvester**
–	Glumes keeled, usually scabrous (at least in part), rarely upper glume smooth throughout, apices obtuse to acute-acuminate, rarely blunt to truncate, rounded to muticous; palea absent or rudimentary to ¾ the length of the lemma; panicles open and diffuse to condensed and spikelike; lemmas usually longer than wide (rarely equally wide as long), usually narrowed towards the apex, apices variable, ranging from somewhat broadly to usually narrowly truncate, usually with 2 to 5 dents (sometimes aristulate), to blunt and entire, awnless or with an awn 0.2–6+ mm long; anthers sometimes longer to usually shorter than the lemma; caryopsis surface usually smooth; perennials or annuals; usually growing from well-developed soils, less often from shallow soils, and generally reliant on soil moisture for their water supply; cosmopolitan	***Agrostis* L.**

## Supplementary Material

XML Treatment for Agrostula

XML Treatment for Agrostula truncatula

XML Treatment for Agrostula truncatula subsp. durieui

XML Treatment for Alpagrostis

XML Treatment for Alpagrostis alpina

XML Treatment for Alpagrostis alpina var. flavescens

XML Treatment for Alpagrostis barceloi

XML Treatment for Alpagrostis setacea

XML Treatment for Alpagrostis setacea var. flava

XML Treatment for Alpagrostis schleicheri
